# Case Report: Delayed Perforation after Definitive Treatment of Focal Intestinal Perforation with a Peritoneal Drain

**DOI:** 10.1155/2012/316147

**Published:** 2012-08-27

**Authors:** Brian G. A. Dalton, Kenneth C. Walters, Melvin S. Dassinger

**Affiliations:** ^1^Spartanburg Regional Medical Center, 101 E. Wood St. Spartanburg, SC 29302, USA; ^2^Arkansas Children's Hospital, One Children's Way, Little Rock, AR 72202, USA

## Abstract

Focal intestinal perforation (FIP) has long been described in the pediatric literature. Peritoneal drainage (PD) is widely used as treatment for focal intestinal perforation. Here we report a premature infant that underwent PD on day of life 9 for a FIP. The infant recovered well from this episode and was discharged home without known sequelae. Subsequently, the same patient presented 16 months later with peritonitis. A perforation was discovered at laparotomy without evidence of surrounding necrosis. Given this finding, we believe this second episode of perforation was at the same site as the initial episode of FIP. The finding of FIP has been described without findings of surrounding necrosis. However, we believe this to be the first report of delayed perforation greater than 1 year from initial presentation after FIP treated definitively with peritoneal drain.

## 1. Introduction

Focal intestinal perforation (FIP) in the very low-birthweight infant (<1500 g) has been a well-described entity for over 20 years [1]. Peritoneal drain has long been an accepted treatment of FIP in very low birth weight infants [2]. These drains have been used initially as a bridge to laparotomy, but when the patient improved after the drain insertion, it became definitive treatment [3]. However, there is a paucity of information on the long-term gastrointestinal sequelae of patients that have undergone peritoneal drainage as a definitive treatment for FIP. Here we report a patient that presented with an adhesive bowel obstruction with concomitant reopening of an intestinal perforation 16 months after definitive treatment by peritoneal drainage. 

## 2. Case Report

**Figure 1:**
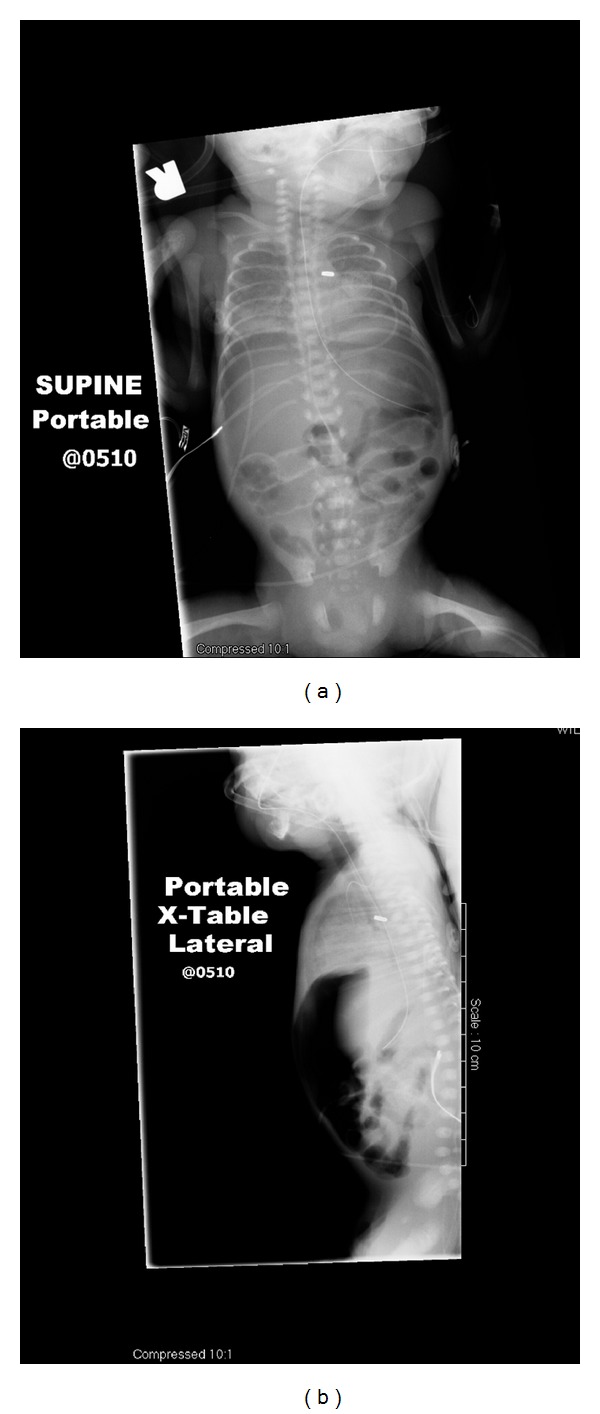
(a) AP abdominal X-ray on DOL 8 showing no portal venous gas or pneumatosis (b) cross table lateral X-ray on DOL 8 showing pneumoperitoneum.

Our patient was born at 26-week gestation weighing 845 grams. Initial echocardiogram demonstrated a large, hemodynamically significant patent ductus arteriosis (PDA); a single dose of indomethacin failed to result in closure, and the PDA was ligated on day of life (DOL) 4. On DOL 9, the patient developed a metabolic acidosis and abdominal distension. Subsequent abdominal radiographs demonstrated the presence of pneumoperitoneum without pneumatosis or portal venous gas (see Figure 1), and a peritoneal drain was placed. The patient's condition significantly improved, and the drain was removed after seven days. Laparotomy was not performed. Of note, the patient had not been enterally fed prior to perforation and drain placement.

**Figure 2:**
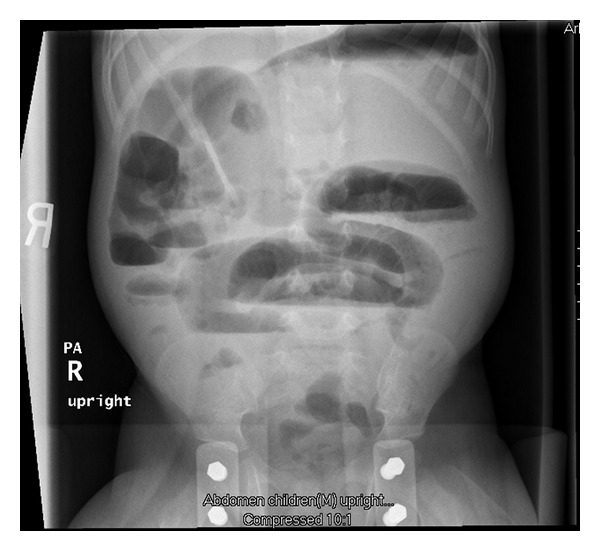
Abdominal XR of JW on re-presentation with obstructive pattern.

Sixteen months after initial perforation the patient re-presented to our facility with abdominal distention, obstipation, and vomiting. Physical exam was consistent with peritonitis and abdominal radiograph is seen in Figure 2. An exploratory laparatomy with lysis of adhesions, small bowel resection and primary anastamosis was performed. An avascular band at the previous peritoneal drain site was found to be causing the obstruction. Interestingly, a perforation was found on the antimesenteric side of the ileum proximal to this obstruction in the face of otherwise healthy, well-vascularized bowel. Pathology was confirmatory, revealing a perforation without any abnormality of the surrounding bowel. Thus, the perforation found at laparotomy was felt to be the site of the initial neonatal isolated intestinal perforation. The patient had an unremarkable hospital course and was discharged on POD 4.

## 3. Discussion

Focal intestinal perforation (FIP) is a much debated topic. Some authors contend that it is a radiologic and histologic distinguishable disease process from necrotizing enterocolitis [4, 5]. Others feel that these disease processes are two ends of the same spectrum [6]. However, certain risk factors seem to be associated with FIP rather than NEC, and our patient had several of these risk factors. First, our patient received indomethacin which has been linked to focal intestinal perforation [4]. Also, no radiographic evidence of NEC (no pneumatosis or portal venous gas) was present in this case, which has been used to differentiate FIP from NEC in multiple studies. Futhermore, the patient discussed here was of extremely low birth weight, born at less than 27 weeks, and was not fed enterally prior to his initial perforation, all of which point to FIP rather than NEC [5–7]. The treatment of this disease has been debated but in extremely low-birth weight infants peritoneal drainage has been shown to be not only a successful bridge to laparotomy but also a definitive treatment [3, 7]. 

The incidence of intestinal stenosis and/or obstruction is well documented after medical and surgical treatment for NEC [8, 9], as often as 30% of the time. Post-NEC strictures or obstructions have also been documented to present as perforation or sepsis [9]. However, incidence of stenosis or obstruction is not well reported after FIP. In fact, we could not find any other reports of spontaneous perforation after FIP treated with PD.

Here we present a case of spontaneous intestinal perforation without previous evidence of NEC stage II as defined by Bell et al. [10] and was diagnosed with pneumoperitoneum four days after the administration of indomethacin. He presented with a bowel obstruction and peritonitis 16 months postop and was found to have an adhesive obstruction and a contained perforation presumably at the previous site of perforation. While we cannot prove definitively that this perforation occurred at the same site as the previous one the pathology report does lend some support to our theory. Final pathology showed normal bowel surrounding the perforation on final path without evidence of surrounding necrosis. An obstructive perforation or one associated with NEC usually demonstrates ischemia and transmural necrosis of the bowel wall surrounding the site of perforation. Similar histologic findings (no ischemia but thinning or absence of the muscle and no evidence of microvascular compromise) have been shown some retrospective studies concerning FIP [11, 12]. However, we believe, this is the first report of such a finding after full recovery from a perinatal episode of FIP. While there does seem to be a trend toward better survival in FIP compared to NEC perforations [13], long-term sequelae of FIP are more difficult to characterize. In recent retrospective study Miserez et al. showed normal GI function at a mean follow up of 23 months without failure to thrive in 14 patients [14]. However, all of those patients underwent laparotomy not peritoneal drain as was the case in our patient. To our knowledge this is the latest a patient has presented with perforation after a spontaneous intestinal perforation definitively treated with primary peritoneal drainage. Furthermore, this is the first report of reopening at a previous site of perforation in a child treated for FIP. This report points out the severe void of studies reporting long term followup in patients with FIP both that underwent PD or laparotomy as a definitive procedure, and the need for such studies.
